# Official Investigation of the Charges Preferred against J. W. Freer, M. D., Surgeon of the Board of Enrollment, 1st District of Illinois, by the Homœopathists of Chicago

**Published:** 1865-03

**Authors:** 


CHICAGO
MEDICAL JOURNAL.

VOL. XXII.] MARCH, 1865. [No. 3.

### ORIGINAL COMMUNICATIONS.

### OFFICIAL INVESTIGATION OF THE CHARGES PREFERRED AGAINST J.W. FREER, M.D.,

*Surgeon of the Board of Enrollment, First District of Illinois, by the Homœopathists of Chicago.*

Considerable curiosity having been manifested in various quarters to ascertain the facts in the case of the trial of Surgeon Freer, on the charges preferred against him with regard to the performance of his duties as Enrolling Surgeon in this district, by the Homœopathic guild of Chicago, we have endeavored, with success, to obtain a copy both of the charges and testimony brought before the Court, which in all their material parts we now lay before our readers. Although from its extreme length it is impossible to publish it *in extenso* in the pages of the *Journal* the reader is assured that no part of the evidence, or facts bearing on the case are omitted.

The specifications against Dr. Freer are contained in the following letter:

Brig.-Gen. Jas. B. Fry, Pro.-Mar.-Gen., U. S. A., Washington, D. C.:—

Sir—The undersigned, a Committee appointed by a fully attended meeting of the Homœopathic Physicians of Chicago, respectfully represent, that the practitioners of our School in this city number more than thirty regularly educated physicians and surgeons, whose diplomas emanate from the most reputable Medical Schools in this country, and whose practice embraces the most wealthy, influential, and enlightened portion of our community.

We therefore claim that we are entitled to the rights and privileges appertaining to the calling, and to protection from our Government against insults and abuse at the hands of its officials.

J. W. Freer, M. D., Enrolling Surgeon of this District, has been guilty of refusing to receive or consider our certificates of disability, while requiring such from the physicians attending the applicants, on the sole ground that they emanate from Homœopathic Physicians, thus in effect forcing the parties to employ Physicians of the Allopathic School, in order to obtain the exemption to which they are entitled. He has embraced the occasion of a presentation of our certificates, to insult both us and our patrons in the most wanton and brutal manner.

He has, in some instances, endeavored to make his official position subservient to his private interests, by making his decisions in favor of exemption depend upon an interested relation of himself to the applicants as patients.

Finally, the said Officer is altogether unacceptable to our community, being personally obnoxious, by reason of his entire ignorance of good breeding, his deficiencies in education, and his consequent unofficerlike and ungentlemanly behavior.

For these reasons, which we pledge ourselves to sustain by abundant and satisfactory evidence, we respectfully request his immediate removal from office.

Committee. { G. D. BEEBE, M. D., Med. Director Staff of Maj.-Gen. Thomas.<br>N. F. COOK, M. D.<br>A. E. SMALL, M. D. }

On the basis of this communication, the Provost-Marshal of the District, Capt. Wm. James, was directed by the Provost-Marshal General, through Lieut.-Col. James Oakes, A. A. P. M. G. of Illinois, to investigate the same and report in full, for transmission to the proper department in Washington.

On the part of the Complainants at the Court of Investigation, held pursuant to this order, were introduced G. D, Beebe, A. E. Small, and N. F. Cook, said to be Homœopathic Doctors. Thos. B. Byrne, C. H. Hudson, W. H. Boyle, L. M. Bennett, Thomas Boyle, probably lay members of the same quasi-medical sect.

*Geo. D. Beebe* being duly sworn, deposed as follows, viz.:

On the 27th day of August, 1864, I gave to Mr. L. M. Bennett a certificate of disability in support of a claim for exemption from draft, and accompanied him to the Board of Enrollment where I introduced him to J. W. Freer, the Enrolling Surgeon, and handed him the certificate, which was accompanied by my affidavit, and set forth the nature of Mr. Bennett's disability. Dr. Freer said to me that he would not receive any certificate signed by a Homœopathic Physician; that they were a set of pretenders and quacks, and that such certificates did not amount to anything. I then called Dr. Freer's attention to the rank I had previously held in the Army, as being in some measure an indication of my professional attainments, and that I could prove to him that I had been educated in the best Schools in the country. His reply was given in a sneering, taunting manner that, "Yes, I know how you got into the Army; you got in there by subterfuge. I found you once sneaking into the Rush Medical College and then kicked you out of the building," etc., etc., statements entirely at variance with the facts in every case, and apparently made for the purpose of insulting me. I then told Dr. Freer that I was not there to defend my character, or to resent insults, that I came to consult him as a government officer. I then asked him if the certificate I had presented was in due form, he took the paper, read it over, and said it was. I then asked him if the facts certified to were sufficient to exempt Mr. Bennett, provided the certificate was satisfactorily signed. He said he supposed they were. I then told him that the qnestion of Homœopathic Physicians being competent to give certificates under oath was by his course, assuming such proportions that we should be compelled to refer it for decision to the authorities at Washington. I cited his attention to the fact that nearly all of these physicians whose certificates he rejected were legal graduates of the very best Schools in the country, such as the *Uuiversity* and *Jefferson* Schools of Philadelphia, and others equally competent to confer the degrees, and if their certificates were sworn to I did not believe the authorities at Washington would discriminate against them. Mr. Bennett then, in my hearing, asked Dr. Freer for a personal examination to determine the question of disability, and Dr. Freer did, in my hearing, refuse to examine him. Mr. Bennett then asked, What am I to do? Dr. F. said, When you can bring the certificate of a regular physician I will attend to you. (Signed,)

Chicago, Oct. 26th, 1864. G. D. BEEBE, M. D.

It is noticeable that, subjoined to this affidavit, Mr. Beebe notes down references to several gentlemen of the city, as vouchers for his professional standing and reputation. As this statement is not sworn to, and as none of the referees are suspected of even a Homœopathic acquaintance with medicine, either as a science or art, we do not find it necessary, or does justice to the Complainants in any wise demand its reproduction in this place.

On *Cross-Examination* Mr. Beebe replied as follows:

In regard to his receiving certificates, I know he said to Bennett that when he could procure the certificate of a regular physician he would then attend to him. In regard to the allegation of making his position subservient to his private interests I know nothing personal, except the certificate of Thomas Boyle, which I herewith submit.

[This affidavit, for convenience, is here introduced.—*Rep.*]

I hereby certify that on or about the 16th day of August, 1864, Wm. H. Boyle and myself applied to the Enrolling Surgeon of this District (Dr. J. W. Freer,) for exemption from draft on the ground of physical disability. The said Wm. H. Boyle, claiming exemption on the disease of the heart, offered in support of this claim, to furnish the certificate of his physicians, Drs. Small and Hale. Dr. J. W. Freer said to my brother, the said Wm. H. Boyle, that he would not take the certificate of any Homœopathic Physician, that such physicians were mountebanks and not capable of treating the sick, but if he would take a prescription, such as he the said Freer would give him, for a few days, he could then tell whether disease of the heart existed sufficient to exempt him. The said Freer did then give my brother medicine with directions for taking the same, and requested him to report to him again in a few days. I further certify that my brother, the said Wm. H. Boyle, did not ask Dr. Freer for medical advice. Dr. Freer also stated to me personally that he would not take the certificate of any Homœopathic Physician. I also certify that my brother, the said Wm. H. Boyle, was called to New York on business two days after receiving the medicine from Dr. Freer, and has not yet returned. (Signed,)

Chicago, Oct. 25th, 1864. THOMAS BOYLE.

Sworn to before Frode Heegaard, Notary Public.

Beebe's testimony on *Cross-Exam.* continued.

*Ques.*—What Medical College were you educated in ?

*Ans.*—In the Albany Medical College and the Homœopath Medical College of Philadelphia.

*Ques.*—Of what School is the Albany ?

*Ans.*—Allopathic.

*Ques.*—Did they call it an Allopathic School ?

*Ans.*—They called it the Albany Medical College. I will not swear that they did not call it an Allopathic School. There are various titles given to such Schools by the people, such as thunder-and-lightning schools. These are merely slang phrases.

*Ques.*—Is or is not it a slang phrase to call it Allopathic ?

*Ans.*—I think not. They are sometimes called the Regular School. I call them Allopathic. I do not know that they regard the phrase Allopathic, as applied to their system, a slang phrase.

*Ques.*—Do you know who were the authors of the application to them of the phrase "Allopathic"?

*Ans.*—The Greeks.

*Ques.*—What is the reputation of the Albany School ?

*Ans.*—One of the best in the country of that class.

Mr. Beebe then stated, in reply to an interrogatory, that he at one time applied to the Ill. State Med. Examining Board for examination as Surgeon, but the request was declined on the ground of being a Homœopathist. [See Dr. Johnson's evidence.]

*Ques.*—Did you afterwards apply to the Board at Washington ?

*Ans.*—I did through Dr. Dyer to the Sec. of War.

*Ques.*—As a Homœopathic or Allopathic Physician ?

*Ans.*—I don't know whether those terms were used or not. They were not in the application. I think Dr. Dyer told me that he informed the Secretary that I was a Homœopathist, and urged my appointment on that ground.

*Ques.*—Were you examined ?

*Ans.*—I was.

*Ques.*—In the theory and practice of medicine, among other things ?

*Ans.*—I was.

*Ques.*—According to what system, as to the theory and practice of medicine ?

*Ans.*—The Allopathic School—the only School with which the Board was supposed to be acquainted.

*Ques.*—Were you requested to write a thesis ?

*Ans.*—I was.

*Ques.*—What was the subject ?

*Ans.*—Acute Rheumatism.

*Ques.*—As to the treatment of that disease in your thesis, did you conform to the Homœopathic or Allopathic treatment?

*Ans.*—To the Allopathic—though I recommended a remedy used in both Schools.

*Ques.*—Did you pass an examination?

*Ans.*—I received the Surgeon-General's certificate that I was recommended by the Board.

*Ques.*—Did the Board, to your knowledge, know you were a Homœopath?

*Ans.*—I don't know. As they had not the appointing power I did not think it necessary to tell them.

*Ques.*—Do you believe they would have given you a certificate if you had told them?

*Ans.*—I have no reason to think they would not.

*Ques.*—Your experience with the Illinois Board is no reason, is it?

*Ans.*—Not at all.

*Ques.*—Do you know a man by the name of Patterson, in this city, and if so, were you called upon to treat him for an injured leg?

*Ans.*—I know Mr. P., and was so called upon.

*Ques.*—What was the difficulty with his leg?

*Ans.*—A comminuted fracture of the patella.

*Ques.*—How, and to what extent was it fractured?

*Ans.*—I discovered six fragments to the patella, giving mobility and crepitus.

*Ques.*—What are the symptoms of fractured patella?

*Ans.*—Those I gave above; the mobility of the fragments, and crepitus.

*Ques.*—Was there any separation of these fragments, and in what direction?

*Ans.*—Owing to the direction of the force which produced the fracture there was but very little separation.

*Ques.*—What forces are always brought to bear in the fracture of a patella, (unless that fracture be longitudinal,) causing a separation of fragments, and to what extent?

*Ans.*—There are no forces always operating to separate the fragments. Usually fracture of the patella takes place while the knee is flexed, in which case the quadriceps extensor femoris, by its contraction, tends to separate the fragments to a considerable extent. In this case the fracture took place while the limb was fully extended. The patient was standing on a box placed on a chair, the man reaching up to the top of a window, the box toppled off from the chair, and as the man fell perpendicularly, his knee struck the sharp corner of the box or the sharp corner of the box struck the patella, a little below the middle, causing the fracture. The contractile force of the quadriceps extensor was not sufficient in this case to separate the fibres of the ligamentum patella, within which the patella is lodged.

*Ques.*—Are there any forces which would cause a depression or descent of the lower fragments?

*Ans.*—Only the slight contraction of the ligamentum patella.

*Ques.*—Of what tissue is that composed?

*Ans.*—Fibrous.

*Ques.*—Which variety?

*Ans.*—Yellow fibrous.

*Ques.*—Is the quadriceps a powerful muscular mass?

*Ans.*—It is.

*Ques.*—Have you not shown that there were two powerful forces acting in opposite directions on these fragments?

*Ans.*—I have shown that there was not, from the fact that the muscle was in a state of contraction, the limb being fully extended at the time of the accident.

*Ques.*—Do not the muscular fibres continue to contract, notwithstanding the points of muscular attachment to bone may have been separated, until they attain an unnatural degree of contraction?

*Ans.*—That is a question I could not answer yes or no.

*Ques.*—Did you change the dressing of Patterson's limb, if so, how long after the fracture, and was your second dressing consistent with your theory of the accident, etc. ?

*Ans.*—That dressing was not changed until some time after, when the man was walking about on crutches, say three or four weeks.

*Ques.*—Did you not place the limb on a double inclined plane, with the limb flexed ?

*Ans.*—I did not. The patient complained at my third or fourth visit that there were muscular jerkings of the limb, and asked if there was not some means by which I could steady the limb. He was afraid he would displace the bandage, I thereupon laid the limb in a Day's fracture splint, still keeping the knee fully extended.

*Ques*—How many cases of fractured patella does Frank Hamilton report as having united by bone ?

*Ans.*—I have not consulted him recently enough to remember.

*Ques.*—Did Patterson's case unite by bony union ?

*Ans.*—I think it did, bony union by means of cartilage.

*Ques.*—Are you acquainted with the public sentiment in regard to Dr. Freer's discharge of his duties as Enrolling Surgeon, exclusive of those who sympathize with the Homœopathic system ?

*Ans.*—Those who do not sympathize with the Homœopathic system are principally foreigners, and I do not know their sentiments.

*Ques.*—How many, exclusive of Physicians, have you heard speak of him as Enrolling Surgeon ?

*Ans.*—I think fifty persons.

*Ques.*—Who were they ?

*Ans.*—I can't tell the names, they are among the prominent and most influential people in the city.

*Ques.*—Can't you name any of them, and do you know whether they had any personal knowledge of the manner in which he discharged his duties ?

*Ans.*—One, I think, was Mr. T. P. Byrne, Mr. James L. Collins, Mr. L. M. Bennett, Rev. Dr. Clark. I have talked with many others, but can't think now. Byrne said that Dr. Freer said a man must be insane or a fool who would employ such men as Dr. Small and Dr. Beebe.

*Ques.*—How many of these men applied for certificates of disability that you have named ?

*Ans.*—I guess all of them applied here at the office.

*Ques.*—Were any of them exempted ?

*Ans.*—Mr. Collins—I don't know about the others.

*Ques.*—Were you examined here for exemption, if yea, how were you treated ?

*Ans.*—I was examined here. The fact was that Dr. Freer did not know me. I was treated very gentlemanly.

*Ques.*—Do you know how long he has been a teacher in Rush Medical College?

*Ans.*—I do not, but I have heard that he has recently left the trade of a blacksmith.

*Ques.*—Who told you so ?

*Ans.*—I don't know.

*Ques.*—Will you tax your recollection to the utmost ?

*Ans.*—I can't tell.

*Ques.*—How long have you been in this city, and has he not been a teacher ever since you came here ?

*Ans.*—I have lived here nearly seven years. I do not know whether he was a teacher when I came here or not.

*Ques.*—Did you not, the first winter you came here, see Dr. Freer in the discharge of his duties as teacher in that College ?

*Ans.*—I think not.

*Ques.*—Did you not enter the dissecting rooms of that College the first winter you came here, and commence dissecting, or was it at a later date?

*Ans.*—I think the second winter that I was here I accompanied a student of mine and made arrangements with Dr. Hollister, my student purchasing a subject from him and paying him for the privilege of dissecting it in Rush College. I accompanied the student to the College and taught him there. I don't know whether I saw Dr. Freer there.

*Ques.*—Do the two Schools recognize each other by consultation, graduating, or otherwise?

*Ans.*—They do. At the time of my pursuing a course of study in the Albany College I told the Dean of the Faculty that I was a student in a Homœopathic office, and asked him if that would make any difference in their conferring the degree of that College upon me. He replied, that need make no difference, or words to that effect. Numerous other instances, both in that and other Schools, have confirmed me in the belief that they are not governed by the individual preferences of students. As regards consultations, I am not infrequently called in consultation by Allopathic Practitioners. One recent instance, was that of a physician about 35 miles out on the NorthWestern Road. He telegraphed for Dr. Beebe or Dr. Brainard to meet him to make an amputation. He was an Allopathic Practitioner of Barrington, Illinois. Dr. Brainard is an Allopathic Practitioner. I met him and performed the operation, and subsequently consulted him in reference to the case, by letter. I might say, in a general way, that a large number of the Practitioners of both Schools recognize each other as members in common of a scientific profession.

*Ques.*—Is it, or is it not, a common occurrence, for Homœopathic Practitioners to graduate at Allopathic Medical Colleges?

*Ans.*—It is. I am personally knowing to the fact that numbers of those who graduated at Rush Medical College in this city, are Homœopathic students, and graduate with the design of practising Homœopathic medicine. The same is true of the Bellevue Medical Hospital of New York city, and numerous other Schools that I might mention. The class at the University at Ann Arbor, Michigan, is sometimes nearly half made up of Homœopathic students.

*Ques.*—About the inclined plane spoken of in the examination, in regard to one Patterson, state whether the limb was placed in inclined planes, and how it came about?

*Ans.*—I stated that the leg was placed in one of Day's fracture splints, and fully extended, but at a subsequent visit I found that the patient had, without my knowledge, slightly flexed the knee. I again extended the limb and left it extended. The leg was never in a double inclined plane by my direction or consent.

*Ques.*—Who was the Allopathic Physician at Barrington who telegraphed to you and Dr. Brainard, and with whom you consulted by letter?

*Ans.*—I can not call his name to mind at this moment.

*Ques.*—Have you conferred with Dr. Brainard to ascertain whether he would not consult with you, and if so, what did he say?

*Ans.*—I did confer with him on that subject, asking him if he would meet me in strictly surgical cases. He said that so far as he himself was concerned, he should have no objection, but he belonged to a School where such course might make trouble with his neighbors. I did, however, with his consent, take a patient to his office, and there consult him in reference to it. The Doctor examined the case, expressed his opinion as to the disease and mode of treatment.

*Qaes.*—Do you know that the Faculty of Rush Medical College are aware that any students are now attending, or have heretofore attended, the College with the intention of practising Homœopathically?

*Ans.*—I do not know, but have presumptive evidence.

*Ques.*—What presumptive evidence?

*Ans.*—The well-known preferences of some students, and their freedom in expressing those preferences.

*Ques.*—What are their names?

*Ans.*—D. A. Colton, I think is one. I believe I will not give any more names.

*Ques.*—Is Mr. Colton a member of the present class, if not, what class?

*Ans.*—No, sir. I could not say of what class.

*Ques.*—Please give the names of those fifty persons who have expressed the opinion you have stated in regard to Dr. Freer as Enrolling Surgeon; how many of them were applicants for certificates of disability; what were their preferences as to the two Schools of medicine, and in your conversations with them, who introduced the subject?

*Ans.*—T. P. Byrn. His preferences are for Homœopathy, and he was an applicant for discharge. I am unable to say who mentioned the subject as to Dr. Freer. James L. Collins was another. He employs both systems in his family. He was an applicant for discharge. He introduced the subject of Dr. Freer to me. L. M. Bennett, the witness who was before the Board; he introduced the subject to me. He was an applicant for discharge. W. H. White is a Homœopathist, not an applicant for discharge. E. Rawson was another, is a Homœopathist, a physician, I think not an applicant for discharge. There are a good many other Homœopathic physicians included, in the city. I am not aware whether they were applicants for discharge or not.

*A. E. Small*, another of the complainants, being duly sworn, deposed and said, that he had no personal acquaintance with Dr. Freer and had no issue with him; that he signed the letter containing the charges as one of a Committee of a Homœopathic Society, supposing that the charges could be sustained. Would not have signed them if he had been aware that the word "brutal" was in the paper. Supposed that he used insulting language to those who differed with him in sentiment only from the statement of others. Relied upon the testimony of Wm. H. Boyle to prove that Dr. Freer made his position subservient to his private interest. Know nothing personally of his deficiency in education or good breeding. This witness further replies as follows:

*Ques.*—How do you regard Hahnemann as a medical philosopher?

*Ans.*—I regard him as a very learned, devoted lover of truth, and one of the most scientific physicians of his time.

*Ques.*—You have read his Organon; do you concur in his treatment of Syphilitic diseases as laid down in the note to the 246th Sec. of the Organon at the close of the section?

*Ans.*—Well, I have never treated them in that way, and therefore am not prepared to say whether I concur or not.

*Note.*—"In pure syphilitic diseases I have generally found a single dose of mercury (X°) sufficient; yet where the least complication with psora was perceptible, sometimes two or three such doses were necessary, given in intervals of six or eight days.

"In those cases wherein a particular remedy is strongly indicated, but the patient is very weak and irritable, once smelling a globule of the size of a mustard seed, moistened with the medicine, is safer and more serviceable than hen it is taken in substance, even in the minutest dose of the higher dilutions. In the process of smelling, the patient should hold the vial containing the globule under one nostril, when one momentary inhalation of the air in the vial is to be made; and if the dose is intended to be stronger, the same operation may be repeated with the other nostril. The operation of the medicine thus administered continues as long as when it is taken in substance, and therefore the smelling must not be repeated at shorter intervals than when taken in the latter mode."

*Ques.*—How does the foot note under Section 288 strike you, as the vagaries of a visionary or the result of sound investigation by a great medical philosopher?

*Note.*—"Homœopathic remedies operate with the most certainty and energy by smelling or inhaling the medicinal aura constantly emenating from a saccharine globule that has been impregnated with the higher dilution of a medicine, and in a dry state enclosed in a small vial. One globule, (of which 10, 20 to 100 weigh a grain,) moistened with the 30th dilution and then dried, provided it be preserved from heat and light of the sun, retains its virtues undiminished, at least for eighteen or twenty years, (so far as my experience extends,) although the vial that contained it had during that time been opened a thousand times. Should the nostrils be closed by coryza or polypus, the patient may inhale, through his mouth, holding the mouth of the vial between his lips. It may be applied to the nostrils of small children while they are asleep, with the certainty of success. During these inhalations the medicinal aura comes in contact with the nerves, which are spread over the parietes of the ample cavities, through which it freely passes, and thus influences the vital power in the mildest, yet most powerful and beneficial manner. All that is curable by Homœopathy may with the most certainty and safety be cured by this mode of receiving the medicine. Of late I have become convinced, (which I would not have previously believed,) that smelling imparts medicinal influence as energetic and long-continued as when the medicine is taken in substance by the mouth, and at the same time that its operation is thus more gentle than when administered by the latter mode. It is therefore requisite that the intervals for repeating the smelling should not be shorter than those prescribed for taking the medicine in a more substantial form."

*Ans.*—It strikes me just in this way: If he was under oath when he wrote it, he was a man of very close observation. It does not read to me as the vagaries of a visionary. I do not see why it should not be the result of sound investigation.

*Ques.*—Are you acquainted with the Rush Medical College by reputation?

*Ans.*—I am.

*Ques.*—Is it an old School of many years standing?

*Ans.*—I believe it is the oldest College in the West.

*Ques.*—What relation does Dr. Freer sustain to the College?

*Ans.*—He is reputed to be a Professor.

*Ques.*—Of many years standing?

*Ans.*—I believe he has, ever since I have lived in this city.

*Ques.*—Do you not know what his standing is as a Physician?

*Ans.*—I do not.

*Ques.*—Did you not live near neighbor to him for several years?

*Ans.*—I lived within one block of him for two years, I think.

*Ques.*—Do you not know his standing as a Physician and teacher in this city?

*Ans.*—I cannot answer from any positive knowledge. I believe he stands very well as a teacher; I have no doubt of it, from notoriety. Well, I think, with those who prefer that kind of practice, I understand that he stands well.

*Ques.*—Did you ever know a Physician who called himself an "Allopathic Physician?"

*Ans.*—Yes, sir.

*Ques.*—Who was he?

*Ans.*—I don't recollect who.

*Ques.*—Was he a Physician of recognized high-standing?

*Ans.*—So far as I know, I cannot say whether he was or not.

*Ques.*—Do you not know that they regard it as an insult to be termed Allopathic Physicians?

*Ans.*—I was not aware of it.

*N. F. Cook*, another complainant, being duly sworn, deposed that he knew nothing of his own knowledge of the charges made against Dr. Freer in the letter to General Fry signed by him. He signed it as one of a Committee.

Cross Examination of Dr. N. F. Cook. *Ques.*—Doctor, do you remember a conversation with Dr. Hurlbut a few months since in this city, at the dining saloon of Ambrose & Jackson, in which you stated to him your motives in commencing your practice in this city as a Homœopathic physician? If so, state the conversation.

*Ans.*—I do not remember any conversation with him on the subject.

*Ques.*—Have you ever had a conversation with Dr. Hurlbut respecting your motives in commencing to practice in this city as a Homœopathist?

*Ans.*—I never have.

*Thomas P. Byrne* and *Charles H. Hudson*, being severally introduced and sworn, deposed, in effect, that while being examined for exemption from the draft, Dr. Freer ridiculed Homœopathic practitioners and their creed. Made no charges of unfairness or bad treatment in any respect or any attempt on his part to make his position subserve his private interest.

*William H. Boyle*, being duly sworn, says: I am 24 years of age, reside in Chicago; am wholesale dealer in fancy goods at 49 Lake street. I have seen Dr. Freer, enrolling Surgeon, once; am not personally acquainted with him. I saw him, say between the 15th and 19th of last August, at his office, corner of Clark and Lake streets. I had been troubled with an affection of the heart for about fifteen months previously. I went there to get examined. I went into his private office, and was there perhaps fifteen minutes. After the examination was over, we came into his front office, and I asked him whether he thought my disease was of such a nature to exempt me from the draft. He said he thought it was caused by indigestion. I told him that could not be the case, as I had been under medical treatment for the past year. I also told him that I had been very moderate in my eating for a long time past, as I was distressed after eating. I also told him that I could bring him a certificate from Drs. Small and Hale, under whose treatment I had been. He answered that would not do, as they were nothing but pettifoggers. I told him I was very sorry to hear that, as they had been our family physicians for a long time. He then wrote me a prescription, which I have in my pocket; I have never used it. I supposed he was giving it to me gratis. He said it would be two dollars, which I paid. I did not use the prescription, as I was under treatment by Drs. Small and Hale. There was nothing further took place. Two days after I left for New York, and I have not taken any medicine since. When he gave me the prescription he gave me directions for taking it. I do not know what was his object. There was no further conversation took place. I did not ask him for the prescription. I believe that when I went into Dr. Freer's office my first words were, "I believe that I have been troubled with an affection of the heart and wish to be examined." After he got through with the examination, I asked him if he thought my disease would exempt me from the draft. When I first went in I went with another man, and he asked for Dr. Freer, who was there. I don't know what conversation took place between Dr. Freer and my friend. After I got through with my examination, I asked him if my disease would exempt me. I said nothing in regard to my wish for exemption until after he got through with the examination.

Cross Examination. *Ques.*—Who went with you to Dr. Freer's?

*Ans.*—A fellow by the name of Edwin Cuyler.

*Ques.*—How did he happen to go with you?

*Ans.*—He was going to be examined for some disease and called at the store.

*Ques.*—Had you a conversation with Drs. Small and Hale, or either, in reference to your visit to Dr. Freer's private office before you went?

*Ans.*—I saw Dr. Hale previous to my visit.

*Ques.*—State all that took place between you and Dr. Hale in refeience to your proposed visit and what he advised

*Ans.*—I asked Dr. Hale whether he thought I had better go to Dr. Freer and be examined. He said he would by all means. That was all he said.

*Ques.*—Mr. Boyle, will you not charge your memory and see if you are not mistaken as to the time when you asked if your disease would exempt you. Was it not after the prescription?

*Ans.*—No; it was previous.

*Ques.*—Do you not know that he made the prescription in the private office?

*Ans.*—No; he wrote it after I came out.

*Ques.*—You have sworn that it was a fellow by the name of Edmund Cuyler who went with you to Dr. Freer's, and your brother Thomas Boyle, has filed an affidavit with the Board that he went with you. Which of you has told an untruth?

*Ans.*—Well, now, I will tell you. Mr. Cuyler came to go with me, I know. I think I went there with ——. I could not say whether I was there once or twice. I have got memorandums—I tell you—Mr. Cuyler, if I could see him. My memory was not good at the time. I had been drinking considerably on that day and previously, and was going to New York, and every thing was in confusion. I admit that my ideas were mixed up.

*Ques.*—Are you willing to swear again that the prescription was given in the front room?

*Ans.*—Yes, sir.

*Ques.*—Are you willing, in consideration of the state of your mind at the time, to swear that you said anything about exemption before you paid the bill?

*Ans.*—Well, I think it probable that I did, as my object was to get exempted. Let me see, now, I have not thought of the thing since the time. Dr. Small came to my store and wished me to be examined. I think it was before I paid the bill that I asked him. Drs. Small and Hale are Homœopathic physicians.

Dr. Freer, the respondent, here admits, for the purpose of saving time, that he has the strongest contempt for the Homœopathic fraternity that words can express, and waives all further proof thereof, and the fact that he entertains such opinions is well-known.

*L. M. Bennett* says: I called upon Dr. Freer personally in this room in August, perhaps the 27th. Asked an examination on a claim of disability. I came alone; made my statement to the Doctor; said I had a certificate from my physician. He asked me then, I think, who my physician was. I told him Dr. Beebe. I can't give the words the Doctor gave me; the amount of it was that he refused to receive the certificate of Dr. Beebe. I asked the Doctor what I was to do; that I was nearly a stranger in town; had fallen in Dr. B.'s hands, and he had been my physician. He said I must get a certificate from a regular Physician. I asked him what I was to understand by a regular Physician. He replied again, "any regular Physician." I believe I asked him then to suggest some name. He said he did not wish to make any distinction between Physicians. I told him then I was acquainted with young Dr. Hurlbut, and either he or some one sitting beside him suggested Dr. Brainard. I think that was about all that occured then. On this occasion I had no certificate. The next and last time I came, Dr. Beebe came with me, and I think had a certificate of my disability, which he handed to Dr. Freer. I could not repeat the conversation that took place between Dr. B. and Dr. F. I can only say that there were some high words between them; I would not pretend to give the words. I do not know any thing different that occurred than Dr. Beebe's statement. When we came in, Dr. Beebe offered the certificate; asked Dr. F. if it was in proper form. Dr. F., after looking at it, said he presumed it was. Dr. B. asked him, will you receive it? I think that was the form. Dr. F. said he would not. Then this other conversation occurred, which I won't pretend to give. I would not think of giving the language further than I have stated. I have heard Dr. Beebe's statement; on hearing, it sounds fresh to my memory, and I believe it to be substantially correct. Not being interested, I did not pay so much attention to it as Dr. Beebe did. After Dr. Beebe went out I remained. I told Dr. F. I did not think I should suffer from the differences between Physicians, and asked him what I should do. He said he had no time to examine such cases, and he, or some one sitting by, suggested Dr. Brainard. I did not call upon Dr. Brainard. I afterward called on Dr. Freer at his office, and he referred me to Dr. Hunt. I do not know any thing further than I have already stated. Dr. Freer, in the first conversation between us, called Dr. Beebe a quack.

Cross Examination.—Dr. Freer, in telling me to get the certificate of a regular Physician, may have said get the certificate of one in good standing. In the interview between Dr. Freer and Dr. Beebe they seemed to be a little excited.

*Ques.*—What did Dr. Beebe term your disease?

*Ans.*—My disease was located in one of my testicles, and he described it by a medical term which I do not remember. The certificate now shown me is the one Dr. Beebe gave me. I see by reference to it that the diseases as described in the certificate are Varicocele of the left testicle and sciatic rheumatism. Dr. Freer did not charge me anything; has said nothing about payment. Dr. Hunt has presented no bill. When I came to Dr. Freer, I told him I thought I had a disease of the kidneys. I think I described some of my feelings, but do not think I mentioned the diseases referred to in the certificate. I think, though, I did mention the sciatic rheumatism. When I told the Dr. my impressions in regard to my disease was the time he told me to be examined by a regular Physician. There was quite a number of persons in the front office. The Doctor said he had not time to examine such cases as mine.

Re-direct.—I heard Dr. F. say in his conversation with Dr. B., "I know how you got into the army; you got in by stealth." I can't recollect whether Dr. F. said he would not receive the certificate of any Homœopathic physician. I did not think Dr. Beebe manifested ill feeling at the time. I think that after a little conversation between them, Dr. F. manifested some feeling; it is not for me to say how much. Dr. Freer made the statement that I have mentioned about Dr. B. stealing into the army.

*Surgeon Freer*, in his defence, introduced the testimony of David Dodge, M. D., J. Adams Allen, M. D., H. A. Johnson, M. D., Daniel Brainard, M. D., V. L. Hurlbut, M. D., Wm. C. Hunt, M. D., I. P. Lynn, M. D., Messrs. Josiah Patterson, Thos. Boyle, Hon. S. A. Goodwin, and I. L. Milliken. This testimony here follows, omitting merely a few repetitions drawn out by cross-examination, and the uniform statements of the medical gentlemen as to the well-known high professional reputation, education, abilities and courteous bearing of Dr. Freer.

*Dr. David Dodge*, being duly sworn, deposed as follows:

TESTIMONY INTRODUCED ON THE PART OF THE RESPONDENT.

*Ques.*—Do you know a man by the name of Patterson living in this city, who was supposed to be suffering with a fractured patella? If yea, who had treated him before you saw the case? Did you examine the knee? If so, how long after the supposed injury; and had there been a fracture of the patella and to what extent?

*Ans.*—I know a man by the name of Patterson in this city. He was injured and the family sent for me, but not finding me at home, sent for Drs. Beebe and Fraser. I went to see the case on my return, and in a few minutes Drs. Beebe and Fraser came in. I wes examining the limb when they came in. They, on coming in, requested me to cease handling the limb, and the family told me then that, being tired of waiting for me, they had sent for Drs. B. and F. I surrendered the case to them. I examined the knee before they came in. The family remarked to me, that, as the Doctor had been to the trouble of going after apparatus, perhaps he had better treat it. I did not find any appearances of fractured patella, other than swelling. I told him I could not see it.

*Ques.*—Suppose the patella were fractured into six pieces, would it be possible that you should not have discovered it in your examination?

*Ans.*—I think I could have detected it very readily.

*Ques.*—Did you see Patterson's limb after the first dressing? If so, how soon after, and how frequently was a change in the dressing, and how long after, and how?

*Ans.*—I think three days after the first dressing, perhaps four. I called to see a lady in the house, and passed through the room. I did not notice at that time whether the dressing had been changed. The next day Professor Allen called to see the lady patient, and I again passed through the room, and looked at the limb. We saw a double inclined plane, Day's splint, standing by the bed. We asked Mr. Patterson what he had been doing with that. He said the Doctor brought it up for him to put his leg on. There was no splint of any kind on the leg at the time; it was secured by india-rubber bands. I saw the leg again six or eight days after this time. I asked Mr. Patterson if he could bend his knee. He said he could, and did do it in my presence. This was ten or eleven days after the accident.

*Ques.*—How long did you examine the knee the first time?

*Ans.*—Perhas one-and-a-half or two minutes.

*Ques.*—Are you sure that it was not fractured?

*Ans.*—I was quite sure at the time and afterwards was positive. I knew it was not, because I could not find any separation into fractures with my fingers.

That was the principal reason. You can always feel the fragments of a patella, there is so little covering, the tissues are so thin.

*Ques.*—Suppose the fracture or separation was no more than the tenth of an inch, could you feel it?

*Ans.*—I can conceive of no such case. They would separate from one to two inches.

*Ques.*—Suppose the patella were broken while the leg was perfectly straight and it was not bent afterwards, would the patella separate then?

*Ans.*—Such a thing is hardly possible, unless it were done by a bullet, and then the muscles would continue to contract and the fragments separate.

*Ques.*—Did you ever know a case where the patella was broken while the leg was straight?

*Ans.*—No, sir. I have seen but few cases in my life, and those occurred while the leg was partially bent.

*Ques.*—How many have you seen?

*Ans.*—I have seen four or five—possibly twice that number; but I cannot remember so many.

*Ques.*—How do you know, then, that the patella would separate if fractured while the leg was straight?

*Ans.*—Because of its muscular attachments; the upper fracture would separate from the lower by the contraction of the muscle.

*Ques.*—Is it wholly impossible that the ligamentum patella should resist the contraction of the quadriceps extensor when the leg was perfectly straight?

*Ans.*—I think it is.

*Ques.*—Would not the periosteum over the patella be sufficient to hold the fractured sections of the patella from separation?

*Ans.*—No more than a piece of wet paper.

### *Dr. J. Adams Allen*, being sworn, deposed:

I reside in Chicago, where I have resided, I think, about five years. I am a teacher in Rush Medical College; Principles and Practice of Medicine. I have taught there ever since my residence in this city. I have been connected with two other medical colleges most of the time since 1848, in the old Indiana Medical College and the University of Michigan.

*Ques.*—Please give me as concise a definition of the word Physician, as you would define the word to your class, say at graduation.

*Ans.*—Well, the term Physician is an old one, and, as its origin suggests, it is intended to include those persons who have studied the laws of nature. As the term is ordinarily employed and is used by ordinary physicians, it is applied to those persons who have studied the laws of nature with reference to health and disease. Technically we call such man a doctor, because he is learned in the laws of nature and health and disease.

*Ques.*—There is a class of persons known as Homœopathists. Are they, to your knowledge, recognized by Physicians in this country as legitimate Physicians, and are they recognized as such? If so, how and to what extent?

*Ans.*—There is such a class, and they are not recognized as Physicians by Physicians.

*Ques.*—Is it customary for Physicians to qualify their identity by any adjunct, such as Allopathy, Eclectic, or other such nomenclature?

*Ans.*—It is not.

*Ques.*—How is such nomenclature as a handle to a Physician's title regarded by the Faculty?

*Ans.*—As an evidence of charlatanry or quackery.

*Ques.*—Are you acquainted with Dr. Freer? How long have you known hm? What is his standing as a teacher, Physician, Surgeon, and as a man, in this community? State your means of knowledge.

*Ans.*—I am acquainted with Dr. Freer. His standing is among the most eminent in each respect. I have been in the habit of daily intercourse with him as a colleague in the College, in medical consultations, in daily intercourse, and know his general reputation.

*Ques.*—Did you see a man by the name of Patterson in this city some months since, said to have had a fractured patella? If you did, did you examine his knee; and if you examined it, did you find any evidences of fractured patella?

*Ans.*—I saw such a man and examined his knee, and found that there was no fracture of the patella,

*Ques.*—How long a time would it require for the reunion of a patella fractured into six pieces, say in a patient of 56 years of age?

*Ans.*—Well, I don't know as I can say. It would depend upon how perfect the apposition of the parts was secured. If the apposition was perfect it might re-unite in six or eight weeks. It would depend on many circumstances. It is difficult to limit the time.

*Ques.*—How would you regard the treatment of such a fracture; the placing the limb in a double inclined plane, the limb one-third flexed?

*Ans.*—I should regard that as aggravating the injury, increasing the displacement, the deformity.

*Ques.*—You are one of the old school are you not?

*Ans.*—I am a Physician. There is but one school.

*Ques.*—Are you one of those called an Allopathic Physician?

*Ans.*—I am what is called such by Homœopathists, not by intelligent people. The term is a nick-name. The word Allopath, as applied to Doctors, is what shyster is to lawyers.

*Ques.*—Do you consider it impossible that a Homœopathic doctor should be a learned man?

*Ans.*—It is impossible for a Homœopathic physician to be an educated man, or an educated man to be a Homœopathic physician. To say "Homœopathic physician" is as great a solecism as to say "black white bird." I ought, perhaps, to use the word "honestly," as an educated man might be guilty of a violation of a moral law.

*Ques.*—How are you in the habit of designating Homœopathic physicians?

*Ans.*—As Homœopathic doctors, or as quacks; or, as we say, clap doctors, Indian doctors, horse doctors, cancer doctors, root doctors.

*Ques.*—Is there not any one of the Homœopathic doctors here for whom you have some esteem and respect?

*Ans.*—Not as a Doctor; as a Physician.

*Ques.*—Are there not some of them who have been educated in some of your Colleges?

*Ans.*—I know of no such instances. It may be, just as there are backsliders from the churches. The children of clergymen sometimes do things their fathers are astonished at.

*Ques.*—Is it quite impossible that a fractured patella should heal in less than six weeks?

*Ans.*—In the case of a man 56 years of age, I should think not. With God all things are possible. I think He did not interfere in this case.

*Ques.*—Did you ever see a case where the parts were in perfect apposition?

*Ans.*—I don't know that I ever did. It is very difficult to get them in such a position and keep them so, without danger to the parts by the pressure. They are never found in apposition immediately after the injury; that is an absurdity. It is impossible. It was never seen on the faee of the earth.

*Ques.*—If the patella were broken while the leg was in a straight position, would not the ligamentous covering be sufficient to keep the parts together?

*Ans.*—No, sir.

*Ques.*—Do you not determine whether the patella is broken by the crepitus?

*Ans.*—I should say not. That is the difficulty in such cases to get the parts together so that there shall be crepitus. It is an absurdity. It is drawn up by the strongest muscle in the body.

*Ques.*—How does Frank Hamilton stand as an authority on that subject?

*Ans.*—Good; one of the best on the subject of fractures generally. I have not examined him on that subject. I can see readily in a longitudinal fracture how there can be crepitus.

### *Dr. Hosmer A. Johnson* deposed:

I am a Physician, Surgeon and Teacher in the Chicago Medical College. I have resided in Chicago fourteen years past. I occupy the chair of General Pathology and Public Hygiene. I was a teacher in the Rush Medical College, I think, for five years.

*Ques.*—Please give the definition of the word Physician as you would define it to your class.

*Ans.*—In its medical sense it is applied to those persons who devote themselves to the structure and action of the human body in a state of health and disease, and to the treatment of diseases by the honest use of all those modes and means that experience and observation has shown to be most useful.

*Ques.*—Is there any association by Physicians with those classes of professed doctors known as Homœopathic doctors, Eclectic physicians, and other similar classes, and is any or either of them in any manner recognized as Physicians by men of your profession?

*Ans.*—There is no such association. They are not recognized as Physicians.

*Ques.*—How long have you known Dr. Freer, and what is his standing as a Teacher, Physician and Surgeon, and what was his position when you first became acquainted with him?

*Ans.*—I have known him fourteen years, a little more perhaps. He was a Physician and Surgeon, and teacher of Practical Anatomy and Surgery in Rush Medical College when I first knew him. His standing is among the first in his profession.

*Ques.*—You heard Dr. Beebe testify that he was examined by the Medical Board at Washington, without disclosing that he was a Homœopathic practitioner; and that in writing his thesis, he gave the treatment of the Physician, instead of that of the Homœpath. (I state the substance of his testimony, and not the language.) Would you regard that course on his part as professionally honorable, or would you consider that he obtained his certificate by subterfuge?

*Ans.*—I did not regard it as honorable, but perfectly in keeping with the action of the Homœopaths with reference to the State Boards. I should consider it a subterfuge. I should consider a man, who would do that, disqualified for a position in the army, under the instructions of the Secretary of War. which requires that a man should have a good moral character.

*Ques.*—Do you know Dr. Beebe?

*Ans.*—I do.

*Ques.*—Don't you think his character is good?

*Ans.*—The only knowledge I have of his moral character, is from the efforts he made to obtain an examination by the Medical Board of this State, and his testimony, which I heard in this room. I judge from those efforts and his testimony that he was not a man of good moral character.

*Ques.*—Did you hear Dr. Allen's testimony as to Homœopathic physicians generally?

*Ans.*—I did.

*Ques.*—Do you agree with him?

*Ans.*—I do wholly, without any qualification.

*Dr. Daniel Brainard* deposed:

I am a Physician and Surgeon, and have been President of the Faculty of Rush Medical College since its organization in 1843.

*Ques.*—Do you know Dr. J. W. Freer? If so, how long have you known him, and what is his standing in the community as a gentleman, teacher, Physician and Surgeon?

*Ans.*—I do know Dr. Freer very well; I have known him sixteen or eighteen years. I should say his standing was good.

*Ques.*—Do Physicians maintain any professional relations with Homœopathic doctors, as such? If so, to what extent?

*Ans.*—They do not maintain any professional relations with them.

Cross-Examination. *Ques.*—Do Physicians never consult with Homœopathic doctors?

*Ans.*—Not unless they violate the rules of their profession.

*Ques.*—Is it a rule of the profession not to consult with them?

*Ans.*—It is.

*Ques.*—Is that a written rule?

*Ans.*—The rule is, not to consult with quacks. They are included in the minds of the professton under that name. I would not undertake to say whether they are named specially. There is a written rule that the profession should not consult with quacks.

*Ques.*—Is that the rule to which you refer where you say, that it is a rule of the profession not to consult with Homœopathic doctors?

*Ans.*—It is.

*Ques.*—Am I to understand you as holding, then, that all Homœopathic doctors are quacks?

*Ans.*—Yes.

*Ques.*—What do you mean by quacks?

*Ans.*—I mean a man who holds himself out to the public for the cure of diseases by some special system, or medicine, different from that used by others.

*Ques.*—Do you never, yourself, consult with Homœopathic doctors?

*Ans.*—Not if I know them to be such.

*Ques.*—Have you ever taken the pains to acquaint yourself with the theory and practice of Homœopathy?

*Ans.*—I have. I have read some of their standard works on the subject.

*Ques.*—What is it that you especially condemn in their theory and practice?

*Ans.*—I condemn the theory on which their system is founded, "similia, &c," and the use of infinitesmal doses.

*Ques.*—Did you ever in your own practice adopt their mode of treatment?

*Ans.*—I never did, except so far as it may be embraced in the doctrines of the profession.

*Ques.*—How, then, can you condemn it without having tried it?

*Ans.*—Because the principle upon which it is founded is contrary to my knowledge of medicines in many cases, and because administering medicines in infinitesmal doses is absurd, and because it has been tested on a large scale by hospital Physicians of eminence.

*Ques.*—Have you read the list of diseases which are given as grounds of exemption?

*Ans.*—I have not particularly read it. I have the regulations; have not read them lately.

*Ques.*—In determining whether an applicant had any of those diseases, would there be any difference between your school and the Homœopathic school?

*Ans.*—I think there would. I think my school would be more competent to determine them than the man who studied Homœopathy.

*Ques.*—Suppose that, instead of studying simply Homœopathy, he had graduated at one of your own Colleges, would there be any difference ?

*Ans.*—I think there would. If he had studied medicine so as to become well informed in it, and then practised Homœopathy, I should think he was not an honest man, and therefore not to be trusted.

### *Dr. V. L. Hurlbut*, being duly duly sworn, says :

My name is V. L. Hurlbut. I reside in Chicago. Am a Physician and Surgeon by profession, of the regular School.

*Ques.*—Do you know Dr. N. F. Cook ? If yea, did you ever have a conversation with him in respect of his motives in commencing practice in this city as a Homœopathist ? If so, when, where, and who introduced it ?

*Ans.*—We had a conversation in reference to a Masonic meeting, in which he invited me to attend a certain meeting, and he said if I failed he would attribute it to our differences in medicine. This was about the last of last February, I think. He introduced the subject in this way. He said they, the Homœopaths, were all right if we would let them alone. He said that they did not confine themselves to infinitesmals, any more than we did to large doses, and that if we would only let them alone we would all come together dove-tail—putting the fingers of both hands together to show the manner. He went into it because he was ambitious and wanted to get a position as soon as he could. He remarked that he (Dr. Cook) had lectured at the Hahnemann College, I think, a day or two before, on Pneumonia; that there were a number of Rush Medical College students present; that these students remarked the similarity of views as to treatment and theory of the disease ; of the two, they, the students, thought he was rather more severe than Dr. Allen in the Rush. This conversation occurred at Ambrose & Jackson's restaurant, perhaps eleven or twelve o'clock at night.

### *Dr. William C. Hunt*, being duly sworn, says :—

I am a physician and surgeon, and reside in Chicago.

*Ques.*—Do you know Dr. J. W. Freer, if yes, how long have you known him, and what is his standing in this community as a physician, surgeon and teacher?

*Ans.*—I know Dr. Freer. I have known him fourteen years past. I think he ranks among the best as a physician, surgeon and teacher.

*Ques.*—Do you occupy an office with him, and have you done so for some time past ?

*Ans.*—Our offices adjoin, there is a door between.

*Ques.*—Do you know what his rule has been as Enrolling Surgeon, in respect to the examination of applicants for exemption, at his private office ?

*Ans.*—He has, I think, universally refused to examine any one there.

*Ques.*—Do you know a man by the name of L. M. Bennett, a witness here, if yea, did he apply to you for medical treatment, and what did he say his difficulty was ?

*Ans.*—I know Mr. Bennett. He applied to me to treat him for disease of the kidneys.

*Ques.*—Did you treat him, and what was his disease ?

*Ans.*—I did treat him. I gave him one prescription. I found no disease of the kidneys. He said he had a disease of the kidneys, and that Dr. Beebe had treated him for it.

*Ques.* How did you arrive at the conclusion that he had no disease of the kidneys, state the test ?

*Ans.*—I examined his urine chemically and microscopically.

*Ques.*—What was the matter with him, if anything ?

*Ans.*—It was a constitutional disease, dyspepsia, or what we would term malassimilation.

*Ques.*—Have you, and to what extent, assisted Dr. Freer in the discharge of his duties as Examining Surgeon, and what has been his treatment to those he has had occasion to do business with officially ?

*Ans.*—I have assisted him for about five or six months altogether. So far as my knowledge goes, his treatment has been uniformly gentlemanly, and according to the regulations.

*Ques.*—How do you regard the position of Examining Surgeon at such a place during the pressure of a draft; is it an easy position, or one of great responsibility and perplexity ?

*Ans.*—I think it one of the most perplexing positions that a professional man can be placed in. It is one that requires a great deal of skill and a great deal of patience and care.

*Ques.*—Did you ever hear him say anything in that connection of Homœopathic Physicians, and if so, what ?

*Ans.*—Yes, I have. That he could not take the statements of those men, nor their affidavits, and other statements in substance to the like effect.

*Ques.*—Did he say why not ?

*Ans.*—He regarded them as mountebanks, charlatans, imposters.

*Ques.*—Did you ever hear him say they were damned old thieves ?

*Ans.*—I never heard him say they were damned old thieves, I have heard him say they were no better than thieves, though not in the presence of their patients. This would be in conversation with me. I can't say exactly when; we have been together so long, and have had such conversations. I can't say whether he ever said this in the presence of others.

*Ques.*—Was he in the habit of using such language ?

*Ans.*—I think he has used it to me several times, I can't say that he ever did to any one else.

*Ques.*—Did you not hear him say that Dr. Small was a damned old thief ?

*Ans.*—I don't know that I ever did, I can't say that he singled out any one of them. I would not like to say that I ever heard him say that of any one of them.

*Ques.*—Did you ever hear him speak of Dr. Small individually ?

*Ans.*—No, sir, I can't remember any instance. I have heard him mention Dr. Small, but can't recall the conversation.

*Ques.*—Did you ever hear him speak of Dr. Cook ?

*Ans.*—In the same way I have heard him speak of Dr. Small, slightingly, of course.

*Ques.*—Did you ever hear him mention any other Homœopaths in the same way ?

*Ans.*—I have heard him speak of Dr. Ludlam, and all the others whose names have been brought before us, in the same way.

*Dr. I. P. Lynn*, being sworn, says :

I am a physician and surgeon, and reside in Chicago. I have practised here since 1851. I have assisted Dr. Freer as Examining Surgeon, at the Provost-Marshal's office, through the months of August, September, and most of Oct.

*Ques.*—Were you present when Dr. Beebe came to the office with L. M. Bennett, if yea, state all that was said between the three parties, and how Dr. Freer conducted himself on that occasion ?

*Ans.*—I was present. Dr. Beebe came in with a gentleman, an applicant for exemption, introduced him to Dr. Freer, and handed the latter his certificate. The Doctor examined the certificate and returned it to him, and stated that he would not be governed in his action by any statements he or any other Homœopath might make, in reference to applicants for exemption. Beebe asked him if the certificate was in proper form. He said yes, that he did not refuse it on that account, that he did not wish to bandy words with him. That was all. The whole transaetion did not occupy the space of more than two minutes.

*Ques.*—State carefully the conduct of Dr. Freer towards those with whom he has been required to do business officially, during all the time you have observed him in this office?

*Ans.*—His conduct has been gentlemanly, courteous, and accommodating. I think he has been very careful and honest.

*Ques.*—Doctor, is there any association with, or recognition of Homœopathic Doctors by physicians, so far as you know?

*Ans.*—There is not.

*Ques.*—Have you been in a position to know the sentiment of this community so far as it has been expressed, as to Dr. Freer's administration of his duties, and have you heard any complaints against him, except by the witnesses that have appeared here?

*Ans.*—I have never heard him censured, except by this class of men, that have made their complaints here. I have heard his conduct spoken of approvingly by various persons who have business to do with the office.

*Ques.*—Who was the man that came in with Dr. Beebe?

*Ans.*—I don't know.

*Ques.*—What disease did he have?

*Ans.*—It is my impression that it was some affection of the Urinary organs, but am not certain.

*Ques.*—Were you in the office when they came in?

*Ans.*—I was in, and was present during the whole interview.

*Ques.*—How were you engaged at the time?

*Ans.*—I was an observer of what was going on.

*Ques.*—Was that all you were doing?

*Ans.*—I did nothing but witness this interview, if I had applicants to examine they waited.

*Ques.*—Are you sure that nothing else was said, than what you said in the direct examination?

*Ans.*—I think nothing else of any importance.

*Ques.*—Are you sure nothing else was said?

*Ans.*—Beebe may have said something else, but Dr. Freer refused to talk with him, and he (Beebe) went out of the room, leaving the applicant in.

*Ques.*—Was nothing else said by Dr. Freer?

*Ans.*—When Beebe called his attention to his rank and title, Dr. Freer spoke saying he was aware of his rank and of the means he took to obtain it. I think Beebe made no reply. That was all he said. I am positive Dr. Freer said but little, as he refused to talk with him. Beebe was decidedly loquacious. Dr. Freer said nothing after he refused to talk with him. I don't know that Dr. Beebe made any remark on Dr. Freer's refusal to converse with him, if he did, it was probably not addressed to him. If he did I did not pay any attention to it. I think Dr. Beebe stated, either as he was leaving the room or before, that he would carry the thing to Washington. I don't think Dr. Freer replied to Beebe at all. I took no part in this conversation.

*Josiah M. Patterson*, a witness, produced and sworn, says:

I am 56 years of age, and reside in Chicago.

*Ques.*—Do you know Dr. Beebe, if yea, did you call upon him to treat one of your knees that had been injured, and when; what did he say was the character of the injury; did he change the dressing after the first dressing, and how, and when, and what was the second dressing?

*Ans.*—I know Dr. Beebe. I called on him to treat my right knee, that had been injured, and he attended it. He said the knee pan was broken into four pieces, patella he called it. He dressed it, putting the leg into a straight position. Three or four days after the first dressing he came again to dress it, and brought with him an apparatus which he called a double inclined plane, and put my leg in it, and bent it in this apparatus so that it was about one-third bent. I

kept it in this apparatus about twenty-five or thirty minutes after he left, when I took it off, the knee pained me so much I could not keep it on. He came to look at it once after that, and said that perhaps it was well enough, if the inclined plane gave me pain, to take it off.

*Ques.*—How long after the injury before you could bend your leg?

*Ans.*—I could bend it, say a week afterward, could raise it up, it pained me to flex it, though not acutely, after the first two or three days.

*Ques.*—Are you sure he placed your limb in a bent position, in the inclined plane, three or four days after the injury?

*Ans.*—I am sure, and it would not exceed three or four days after the injury. He said it would relieve it to place the limb in that position.

Dr. Beebe dressed the leg in company with Dr. Fraser. Dr. Fraser is a Homœopathic Physician, in company with Dr. Blanchard. By turning my head I could see my foot while it was in the plane, though I don't recollect that I looked at it, it was not attractive. I took especial notice how my leg was dressed. I noticed how it was dressed in the first place. It could not be changed a quarter of an inch without my knowing it. I am something of a mechanic and notice such matters.

The testimony of Hon. S. A. Goodwin is omitted, as it is merely with reference to Surgeon Freer's general and professional reputation.

*Thomas Boyle*, being sworn, says:

I am 27 years of age, and am a merchant, residing in Chicago.

*Ques.*—There has been an affidavit filed with the Board in this matter, purporting to have been made by you, did you ever make that affidavit?

*Qns.*—I did.

*Aues.*—You state in that affidavit that about the 16th of Augnst, 1864, your brother, Wm. H. Boyle, and yourself applied to the Enrolling Surgeon in this District (Dr. J. W. Freer,) for exemption from draft, is that affidavit true in that respect?

*Ans.*—I applied myself, my brother did not go with me.

*Ques.*—Who presented this affidavit to you, to swear to?

*Ans.*—Dr. Hale, partner of Dr. Small.

*Ques.*—Who drafted the affidavit?

*Ans.*—I don't know.

*Ques.*—You swear that your brother applied for exemption on account of disease of the heart, how do you know if you were not present?

*Ans.*—I only know from what he told me. All the statements in that affidavit so far as they apply to him, I only know from what he told me. In the affidavit, where I am made to say that my Brother did not apply to Dr. Freer for medical advice, I know nothing on the subject except what he told me. I only know from what he told me that Dr. Freer requested my brother to report to him in a few days.

*Ques.*—Did you know that you were swearing in that affidavit to a series of alleged facts of which you could have no personal knowledge?

*Ans.*—I did not.

*Ques.*—Did Dr. Hale request you to sign and swear to that affidavit?

*Ans.*—Yes, sir.

*Ques.*—Did he know that you had no personal knowledge of the facts therein recited, so far as they related to the interview of your brother with Dr. Freer?

*Ans.*—He did. I expressly told him. He said he wanted it so as to get a continuance until my brother returned.

*Ques.*—Did you suppose there was anything in the affidavit which showed of itself that it was stated on hearsay?

*Ans.*—I so stated to the Notary at the time he read the affidavit. I don't know whether Dr. Hale read it or not.

*Ques.*—Did Dr. Hale say anything to indicate that he supposed the affidavit to be hearsay?

*Ans.*—Yes. I remarked to him that it would not be good for anything because it was hearsay.

*Ques.*—What reply did he make?

*Ans.*—He said he only wanted it for a continuance until my brother arrived.

*Ques.*—Did you mean to say in that affidavit that you and your brother went together, or that you went?

*Ans.*—Only that we went.

*Ques.*—At your first conversation with Dr. Hale relative to this affidavit, did you tell him substantially the same story as is contained in the affidavit?

*Ans.*—Yes, sir, and also said it was only hearsay.

*Ques.*—Did you tell him that you and your brother went together?

*Ans.*—No, sir.

*Ques.*—Did you tell him that all you knew of the affair, as it related to your brother, you derived from his statements?

*Ans.*—I did.

*Ques.*—The second time he called on you did he bring the affidavit with him, and did you go with him to a Notary to swear to it?

*Ans.*—He brought the affidavit with him, and I went with him to a Notary.

*Ques.*—When you swore to that affidavit of positive statements, did you tell him and the Notary that as to all the statements relating to your brother, you only knew them by hearsay?

*Ans.*—Yes, sir. The Notary made no reply that I know off; Dr. Hale handed me the affidavit and I read it over. I made no objections to signing it when at the Notary's. At the store I told him it was only hearsay evidence.

*I. L. Millikin*, being duly sworn, says:

I am 51 years of age. I am Enrolling Commissioner for this District. I have resided in this city for 27 years.

*Ques.*—Have you been Mayor thereof?

*Ans.*—I was in A. D., 1854.

*Ques.*—Do you know Dr. Freer, Enrolling Surgeon, if so, how long have you known him, and what is his standing as a physician and surgeon and gentleman?

*Ans.*—I know Dr. Freer, have known him, I think, about twenty years. I have always supposed his standing to be good in all these respects. As it respects his standing in his profession I have heard it spoken of as eminent.

*Ques.*—Have you observed the manner in which he has discharged his duties as Examining Surgeon in this District, if so, how, and your means of knowledge?

*Ans.*—I have been associated with him in this business, so that I have had an opportunity to observe his manner. I think he has been very attentive, careful and discreet in his examinations. As a general thing I have considered him kind and courteous to those he has had to examine.

It is, perhaps, unnecessary to state that the Provost Marshal of the district, after hearing the testimony in the case, reported to the proper bureau in Washington, that "the charges were wholly without foundation," and that, in his opinion, "they were groundless and unworthy of note." Furthermore, that in the discharge of his official duties, "Dr. Freer had been always found courteous, kind and obliging," exercising a "most remarkable forbearance," although oftentimes annoyed by the importunities of those seeking exemption from the draft without reasonable cause or excuse therefor.

Tedious in detail as the case may seem to the casual reader, it is nevertheless one which should attract the attention of every lover of scientific medicine. With the exception of the brazen attempts of homœopathists to foist themselves into the Medical department of the army, (one striking instance of which is developed in the foregoing testimony,) there has scarcely ever been a bolder effort upon the part of schismatics to bring themselves into official notice. From the evidence it is seen that at least one homœopath succeeded in securing a temporary position in the military service by concealing his real opinions, and he has the effrontery to claim his successful fraud as proof of his own, to be presumed respectability! Does the reader wonder that Surgeon Freer distrusted a certificate signed by one who unblushingly swore to his own shame?

The leader of the onslought upon Surgeon Freer was one G. D. Beebe, who treated Josiah Patterson for comminuted fracture of the patella, six pieces! Look now at the testimony on that point and see whether G. D. Beebe, who hid his homœopathic proclivities from the National Examining Board, is surgeon enough, or honest enough, even supposing other qualifications abundant, to have his certificate of disability treated with a grain of respect. The Federal Constitution prevents us adding a word to the record: "Cruel and unusual punishments shall not be inflicted."

The next complainant and witness is one A. E. Small, a person who strenuously insists that he does not know Dr. Freer, (although six years ago, in an advertising sheet scattered indiscriminately in the door-yards and barn-yards of the city, in juxtaposition with Lock hospital and "Dr. D.'s" circulars, he publicly addressed him in a series of scurrilous and blackguard articles.) This man testifies that he knows *nothing personally of the matters alleged.* He *knows* nothing except that he is one of a committee of the Homœopathic fraternity. *He* is only a kitten whose paws that monkey uses to pull nuts from the fire. He swears that he regards Hahnemann as "a very learned and devoted lover of truth, and one of the most scientific physicians of his time"—smelling bottle and all especially endorsed! Does not call himself a Homœopath—*lucus a non lucendo*—but is a Professor in a Hahnemann college and an compiler of a Homœopathic book. He swears that "Allopathic" students are instructed in all the branches of the scientific curriculum, but paternally adds that he thinks "if they would only study Homœopathy they would be greatly benefitted." Works of fiction have been recommended by many abler men than even Mr. Small as an addition to the harsh and dry reading of earnest students.

Let it be put on record that this man Small *swears* he never was a "Botanic" doctor in the State of Maine. Our previous sympathy for the State of Maine is to this extent ameliorated. The city of Philadelphia, which for many years was blessed by his presence, and the infinitesimal college therein, (long since gone into hopeless desuetude,) still remain to give us room for melancholy.

N. F. Cook swore that he knew nothing—that he was only a Homœopathic committee-man. How Homœopathic *he* is, the evidence of Dr. V. L. Hurlbut abundantly discloses. N. F. Cook is noticeable only as a variety of a species, and there are but two species in the genus. One is ignorant, the other knavish. The varieties are mixtures.

One man who kept clear of the committee and clear of the witness stand, the testimony of Thomas Boyle shows to have been named "Dr. Hale." This man Hale, the witness swears, presented him a carefully drawn affidavit to sign and swear to, knowing that he (Boyle) had no personal knowledge of the alleged facts. He (Hale) stated in reply to witness' conscientious scruples to swearing to mere heresay evidence, that it was "only to procure a continuance." This man Hale brought an affidavit containing what the witness himself admits to have been falsehoods, and induced the witness to swear and sign it, "to procure a continuance!" The atrocity of the act carries its own comment.

Sworn to as positive—sworn to as heresay only! The position of "Dr. Hale"—he may perhaps consider an enviable one—we only place him by the side of *Small* (in the comparative degree) and say *Par nobile fratrum.*

The "lay" evidence in this case on the part of the complainants is too contemptible, too frivolous, and, on the part of the witness most depended upon, too thoroughly mixed with very bad whiskey to need remark.

The attack upon Surgeon Freer, trumpeted by all the Lilliput of medicine as one which was to overwhelm him, and with him legitimate medicine, has not only failed but shown itself an abortion begotten by Folly upon Fraud and sent into this breathing world not even half made up.

Chicago, it may be mentioned to country readers, is a peculiar city. It has been the rendezvous of men of desperate fortunes and uncertain morals, as well as of men of of enterprise and far-seeing sagacity. The Ishmaels whose hands are against every man are abundant. In the lottery of chances incident to the growth of a great city many men of limited capacity and even gross ignorance have been thrown into notice because of their accidental wealth. Men of this cast, and especially their wives, are addicted to the wildest, the absurdest follies. Among their pet absurdities, Homœopathy is one of the most prominent. "Down East" doctors "gone to seed" at home come here and pander to these *parvenues* by titillating their palates with infinitesimals, and to their self-complacency by the arts of the sycophant. *Parvenu* "doctors," just loose from some Homœopathic college, (God save the mark!) please the fancy of shoddy goddesses by their neck-ties, dancing-master airs and equipages. Shoddy is triumphant, and infinitesimals rampant. "It is *so nice* and so much the thing, you know, to take sugar plums and pellets rather than gross mixtures!"

We have only to say to those of our readers who hear that Chicago is homœopathic, that the physicians of this city in good and lucrative business number at least ten times the quacks of the homœopathic persuasion or practice. Even "shoddy" is beginning to be nauseated with its pellets and dilutions. The immense majority of the intelligent citizens of Chicago adhere to the regular profession and scorn the knavery of the infinitesimal charlatans with a depth of feeling quite equal to that entertained by the present writer, which, it may be explained, approximates as nearly as possible the sentiments of Surgeon Freer.

